# Romanian Version of the Oral Health Values Scale: Adaptation and Validation

**DOI:** 10.3390/medicina58040544

**Published:** 2022-04-14

**Authors:** Beatrice Adriana Balgiu, Ruxandra Sfeatcu, Christina Mihai, Mircea Lupușoru, Mirela Veronica Bucur, Laura Tribus

**Affiliations:** 1 Department of Career and Educational Training, University Politehnica of Bucharest, 313 Splaiul Independenţei, 060042 Bucharest, Romania; beatrice.balgiu@upb.ro; 2 Department of Oral Health and Community Dentistry, Faculty of Dental Medicine, “Carol Davila”, University of Medicine and Pharmacy, 17–21 Calea Plevnei Street, 010221 Bucharest, Romania; 3 Department of Preventive Dentistry, Faculty of Dental Medicine, “Carol Davila”, University of Medicine and Pharmacy, 17–21 Calea Plevnei Street, 010221 Bucharest, Romania; christina.mihai@umfcd.ro; 4 Department of Physiology, Faculty of General Medicine, “Carol Davila”, University of Medicine and Pharmacy, 8 Eroilor Sanitari Boulevard, 076241 Bucharest, Romania; 5 Department of Prosthesis Technology and Dental Materials, Faculty of Dental Medicine, “Carol Davila”, University of Medicine and Pharmacy, 17–21 Calea Plevnei Street, 020021 Bucharest, Romania; mirela.bucur@umfcd.ro; 6 Department of Internal Medicine, Faculty of Dental Medicine, “Carol Davila”, University of Medicine and Pharmacy, 17–21 Calea Plevnei Street, 020021 Bucharest, Romania; laura.tribus@umfcd.ro

**Keywords:** health values, OHVS, validation, Romanian adults

## Abstract

*Background and Objectives*: Oral health values are an indicator of how people decide their priorities for oral health—an integral part of general health. The purpose of the study was the validation of the Oral Health Values Scale (OHVS), which measures the extent to which individuals invest in their oral health and which includes four factors: Professional dental care, Appearance, Flossing, and Retention of teeth. *Materials and Methods*: A cross-sectional study was conducted on a sample of adults (*n* = 869; Mean age = 32.98) who completed the online questionnaire set. The OHVS was translated into Romanian through the forward-backward translation procedure. The construct validity was assessed through a confirmatory factor analysis (CFA) based on the maximum likelihood estimation method. The convergent validity was assessed by associating the OHVS with scales that evaluate the oral health quality of life (OHIP-14), the attitude towards one’s dentist (R-DBS), oral self-care (DNS), and general health literacy (GHL). The internal consistency was examined using Cronbach’s α and McDonald’s ω. *Results*: The CFA supports the four-factor model of the scale. The OHVS total score and its subscales are negatively associated with the impact of oral health on the quality of life (except for the Appearance subscale) and with a distrust in the dentist’s treatments; they are positively associated with oral self-care and general health literacy. The gender difference shows females have higher scores on all four subscales. The internal consistency is good for Appearance, Flossing, and the OHVS total score, but weak for Professional dental care and Retention. *Conclusions*: OHVS is a valid instrument in the Romanian context that can enrich the set of tools that contribute to oral health research, especially in epidemiological studies.

## 1. Introduction

The state of oral health is determined by a complex of interactions between socioeconomic status, beliefs, behaviors [1,2], values [3,4], and oral health literacy [5].

The term oral health values is defined as the importance the individual gives to self-care and to their trust in professional care [4,6]. The notion of oral health beliefs represents the individual perceptions of the ability to control the results of one’s own oral health [7]. A positive perception of these abilities represents increased care priorities [8]. Thus, knowledge and education contribute to this. Research shows that oral care education is associated with beliefs in the highest oral health level [5,9]. In terms of process, beliefs often have an intuitive and unreasonable character. Values require a process of reflection, of analysis. Awareness of one’s own values depends on knowledge, on oral health literacy—the degree to which individuals have the ability to understand basic oral health care information [10].

For the evaluation of oral health beliefs, there are a series of instruments, such as those based on clearly established patterns (Health Beliefs Model), for example, the Oral Health Belief Questionnaire, which measures five types of beliefs regarding oral health: perceived seriousness, benefit from preventive practices, benefit of plaque control, effectiveness of dentists, and perceived importance [11], validated in various contexts [12,13]; the Oral Health Behavior Questionnaire for Adolescents, which takes into account the adolescents’ beliefs about oral health behaviors [14]; or tools that assess oral health knowledge, attitude, and behavior (for example, the KAB questionnaire), thus linking oral health literacy, oral health beliefs, and behaviors in a single tool [15].

However, for the assessment of oral health values, the tools are reduced to a considerable extent. In the literature, it was considered that the Oral Health Impact Profile (OHIP-14), which considers the poor impact of oral health on the quality of life [16], is a tool that measures oral health values [17]. However, it is worth mentioning that oral health, in turn, depends on how much the individual values it. The first tool built on this approach, namely, what the patient values, is the Oral Health Values Scale (OHVS) [4], which takes into account the variation of values and helps explain the differences in using oral care and treatments.

The OHVS is a multidimensional instrument built in 2021 whose purpose is to identify and measure the values of one’s oral health [4]. Unlike other instruments that deal with oral health (OHIP-14 is focused on the quality of life) [16] or the Hiroshima University-Dental Behavioral Inventory on oral self-care behavior (HU-dbi) [18], in the OHVS, the focus is on how much attention an individual pays to oral health care and on how much one invests in auxiliary oral health care. The validation of the scale was conducted in a case of American respondents. The scale is composed of twelve items, of which six are reversed, assessed on a continuum from 1—strongly disagree to 5—strongly agree. The total score of the scale is between 12 and 60 items. The items are distributed within four factors:

1. Professional dental care and the costs associated with it in terms of energy, time, and focus (e.g., Going to a dentist is not worth the cost to me).
2. Appearance and health aims to consider dentition as a source of pride and the evaluation of oral health as part of general health (e.g., My smile is an important part of my appearance).
3. Flossing as a result of consistent self-care behavior (e.g., Flossing my teeth every day is a high priority for me).
4. Retention of natural teeth reflects the values of oral health by means of the implications it has in one’s functioning and personal health (e.g., It is important to me to keep my natural teeth).

The internal consistency reported on by the authors in the case of the sample of American subjects is good, both for each and every subscale (αs < 0.70) and for the entire instrument (α = 0.84). The association of the OHVS with instruments that evaluate constructs of oral health provided the evidence for concurrent and convergent validity. In the development and validation stages, the OHVS was associated with various instruments that measure the oral health impact profile (Oral Health Impact Profile-14—OHIP-14), distrust of dentist (Revised Dental Beliefs Survey—R-DBS), oral health literacy (Health Literacy in Dentistry Scale—HeLD and Comprehensive Measure of Oral Health Knowledge—CMOHK), oral self-care (Dental Neglect Scale—DNS and Importance of Oral Self-Care Behavior—IDB), and dental anxiety and fear (Dental Fear Survey—DFS and Index of Dental Anxiety and Fear—IDAF-4C) [4]. A similar pattern of the OHVS relationship with the other instruments was observed in both stages. Thus, the OHVS negatively correlates with the quality of life related to oral health, dental fear, and distrust of dentist. It positively correlates with oral health literacy and oral self-care.

The purpose of the present research is to adapt and validate the Oral Health Values Scale (OHVS) into the Romanian language and test the factorial structure and the evaluation of the psychometrical properties of the scale.

The rationale for this approach was motivated by the fact that the OHVS is the first instrument of its kind in the assessment literature that deals with the evaluation of the values related to oral health and with the evaluation of the doctor–patient relationship. Secondly, we were motivated by the fact that researchers and clinicians need valid instruments regarding the concepts that are important within one’s culture and language for epidemiological studies and oral health education sessions in dental offices. These studies may put forth a profile of the population in various social environments with regard to their personal values concerning health, an important aspect given that the latter influences one’s lifestyle. One’s healthy behavior regarding one’s cariogenic diet, personal oral hygiene, and dental visits can be changed according to the values concerning health within programs meant to promote health in communities, with tailored oral health education messages.

Upon reading the study of its development and validation published in 2021, one can infer that the OHVS has good psychometric properties [4]. However, in Romania, people do not go to the dentist as often as the people in other countries in the European Union do, although medical services in private practices are financially acceptable [19,20,21]. In addition, 18% of a population of 5000 Romanians, investigated in 2021 by the National Inspectorate of Public Health, had not visited the dentist in the last 5 years [22]; therefore, there could be differences with regard to the degree to which individuals invest in their oral health. Populations from different countries can have different perceptions on what good oral health means.

### Hypotheses

Considering the available literature [4], the authors propose the hypothesis that the four-factor OHVS is structurally valid as shown by confirmatory factorial analysis (CFA). In addition, the authors expect to obtain significant associations between the OHVS and the tests that measure other oral health constructs: Oral Health Impact Profile (OHIP-14), Dental Neglect Scale (DNS), Revised Dental Beliefs Survey (R-DBS), and general health literacy (GHL).

## 2. Materials and Methods

### 2.1. Ethical Considerations

The study was conducted in full accordance with ethical principles, including the 1975 World Medical Association Declaration of Helsinki as revised in 2000 and 2013. The study was approved by the Ethical Commission of the University of Medicine and Pharmacy, Bucharest (Protocol No. 28447/18 October 2021).

### 2.2. Linguistic Adaptation of the OHVS into Romanian

The adaptation of the scale to the Romanian language was made using the forward-backward translation procedure, following the recommendations put forth in the domain of the adaptation of instruments for cross-cultural research [23]. Thus, there were several stages: 1—the scale was translated into Romanian by two different dentists who speak English fluently, one who is an author of the present study and the other a dentist who practices medicine in the US and is bicultural (familiar with both Romanian and American culture); 2—the two versions were compared, and a synthesis was generated; 3—a blind backward translation of the initial version was made by three other fluent English speakers, two dentists who are authors of the study and an experienced researcher in the social domain; 4—the translations were compared, and a new synthesis (the final version) was generated; 5—the preliminary testing in which the instructions, items, and format of the instruments were evaluated was performed on a sample of 20 adults between the ages of 25 and 31 who were asked to make assessments on the clarity and difficulty of understanding the items. According to the recommendations, a pilot study was performed with people from the population in which the instrument is to be used (in the case of the study, adults), having between 10 and 40 people [23]. These were not included in the final sample on which the evaluation of the psychometric properties of the OHVS was performed. This pretesting did not cause any changes in the established version.

### 2.3. Participants and Recruitment Procedure

The data were collected between the 20 October and the 27 November 2021 by distributing a Google Forms link with the research instruments via social networks, email campaigns, and WhatsApp. The link had an introductory text that explained the objective of the study, i.e., the fact that participation in the study would not involve risks and that no rewards would be given. Moreover, it was disclosed that the respondents would give their consent for the use of the data provided in order to achieve the objective of the study, while maintaining the confidentiality and anonymity of the information. The eligibility criteria were a minimum age of 18 years and the respondents’ Romanian residence. In order to avoid the specific errors of online studies, the questionnaire was secured so as to allow its completion only once by a person who received the link from an official address, and the link was accessible to each person for only 3 days. The participants were informed that all the answers would be used exclusively for scientific purposes, according to the EU Rules and Regulations 2016/679 concerning the protection of natural persons with regard to personal data processing. At the same time, the respondents gave their informed consent with regard to data security. As a control procedure of the biasing methods, the tools were completed anonymously [24].

### 2.4. Instruments

#### 2.4.1. Oral Health Values Scale

The OHVS measures the relevant domains of the values regarding oral health [4]. The scale is made of 12 items assessed on a scale from 1—strongly disagree to 5—strongly agree, and it contains four factors with 3 items each: Professional dental care, Appearance and health, Flossing, and Retention of one’s natural teeth. The OHVS authors report Cronbach internal consistency coefficients over 0.70 in the case of subscales and 0.84 for all 12 items [4].

#### 2.4.2. Oral Health Impact Profile OHIP-14

The OHIP focuses on the quality of life related to oral health [16,25]. The questionnaire that measures the individuals’ perception regarding the social impact of oral disorders on one’s personal wellbeing has 14 items (assessed from 0—never to 4—very often) included in seven dimensions, each of them with 2 items: functional limitation, physical pain, psychological discomfort, physical disability, psychological disability, social disability, and handicap. The internal consistency for the total score in various cultural models highlights coefficients over 0.90. The scale was validated on a sample of Romanian adults and proved to have good psychometric properties [26]. The total score was calculated using the additive method. For this study, the Cronbach coefficient for the total score is 0.932 (95%CI—0.926–0.939), while the CFA shows an acceptable construct validity χ^2^/df = 4.90; CFI = 0.96; RMSEA = 0.070; SRMR = 0.038.

#### 2.4.3. Dental Beliefs Survey-R—R-DBS

The R-DBS measures the patients’ attitude and opinion about the dentist and dentistry services [27,28]. The adaptation and the validation of the 28-item instrument, assessed on a 5-item scale (ranging from 1—never to 5—nearly always) on a sample of Romanian adults, led to four factors: Professionalism (9 items), Comfort (9 items), Communication (7 items), Implication (3 items) [29]. All statements are positive, and the score of the items for each factor is summed. In the case of the present study, the Cronbach coefficient for the total score is 0.965 (95%CI—0.961–0.968), and the CFA shows acceptable goodness-of-fit indices: χ^2^/df = 4.08; CFI = 0.94; RMSEA = 0.060; SRMR = 0.037.

#### 2.4.4. Dental Neglect Scale—DNS

The DNS is used to measure the adults’ behaviors and attitudes regarding oral health [30,31,32]. The scale contains 6 items (one is reverse—[31]) assessed on a continuum from 1—totally disagree to 5—totally agree, and it is unifactorial. The Cronbach coefficient of the instruments reported by the authors is 0.71 [30,32]. For the present study, the scale was translated from English into Romanian using the back-translation procedure, following the guide for cross-cultural research [23]. The translation from English into Romanian and the reverse were done by dentists fluent in English. For the present research, Cronbach’s α is 0.700 (95%CI—0.667–0.729), and the CFA shows good fit: χ^2^/df = 2.48; CFI = 0.99; RMSEA = 0.041; SRMR = 0.019.

#### 2.4.5. Single-Item General Health Literacy Scale—GHL

General health literacy was assessed by using a screening question on a 5-point Likert scale, inspired by the study of Chew et al. [33] and considered efficient in the detection of adequate–inadequate health literacy: How often do you have someone help you read hospital materials? This single question can rapidly identify 80% of adult patients with inadequate health literacy. The ROC analysis results highlight an AUC value of 0.87 (95%CI 0.78–0.96) [33].

### 2.5. The Sociodemographic Data

The collected data are related to (i) self-identified gender, (ii) age, (iii) studies (primary studies, secondary studies, university studies, post-university studies), (iv) domicile (urban versus rural), (v) work sector (public, private, another), and (vi) the geographical region (the main eight regions of the country were included).

### 2.6. Data Analysis

The main strategy of data analysis was the CFA: “a type of structural equation modeling (SEM) that deals specifically with measurement models” [34]. The model fit was assessed by means of the following goodness-of-fit indices: χ^2^/df (criterion chi-squared/degrees of freedom), the lower, the better, preferably <3 [35]. Since χ^2^ is sensitive to sample size, a combination of indices was used, according to the recommendation of West et al. (2021) [36]: CFI (comparative fit index); TLI (Tucker–Lewis index); IFI (incremental fit index), whose optimal value should be ≥0.95 [37]; RMSEA (root mean square error of approximation) and SRMR (standardized root mean square residual), which have good values of approximately 0.06 [38]. AIC (Akaike’s Information Criterion) was used for the comparison of the models. A lower value shows a better model fit [37]. The internal consistency was assessed by means of Cronbach’s α and McDonald’s ω coefficients, considered fit even when they are over 0.60 [39]. Convergent validity was performed by the association between the OHVS and other validated scales that measure other oral health constructs (OHIP-14, DNS, R-DBS, general health literacy). The difference of the average values for the gender criterion was calculated with the Mann–Whitney U test. The discriminant validity was verified by the Fornell–Larcker criterion [40]. The data were analyzed with the programs SPSS22, Amos 22 (IBM, New York, NY, USA), and JASP 0.14.1 (University of Amsterdam, Amsterdam, Netherlands). The latter was used to calculate McDonald’s ω.

Before analyzing the factorial structure, the possibility of the respondents’ social desirability was taken into consideration. In order to do this, the post hoc procedure for the examination of the common method variance (CMV), Harman’s single-factor test [24], and the procedure of the correlation matrix were used [41]. In this regard, an exploratory factorial analysis (EFA) was carried out with the aim of verifying the CMV. All the items for every construct in the study were included in a factorial analysis, and the principal component analysis (PCA) was run.

## 3. Results

### 3.1. Sociodemographic Characteristics of the Sample

The data collection resulted in a convenience sample of 869 respondents with an average age of 32.98 (S.D. = 14.09; the youngest respondent—18 years; the oldest respondent—75). The sample contained a higher proportion of female subjects—618 (71.2%; Mean age = 31.17; S.D. = 14.03)—than male subjects—251 (28.8%; Mean age = 29.51; S.D. = 14.47). As for their education, most participants were university graduates (46%), followed by participants with secondary education (23.1%) and with postgraduate studies (21.6%). The most underrepresented were individuals with primary/elementary studies (9.3%). Most respondents came from the urban environment (83.7%). As for the domain of work, 31% worked in the public sector, 40% worked in the private environment, and 29% were unemployed.

### 3.2. Factorial Structure

The possibility of the occurrence of social desirability in completing the tools was analyzed through the common method variance examination through EFA (unrotated factorial solution) [24]. The results showed eight distinct factors that made up 62% of the total variance (Kaiser–Meyer–Olkin = 0.89; Bartlett’s test of sphericity = 31,916.94; df = 1770; *p* = 0.000). There was more than one factor in the factorial matrix, and the first factor did not capture the largest part of the variance [42]. In addition, the CMV was demonstrated using the procedure of the correlation matrix, which showed correlations between constructs <0.90 [41]. Therefore, it can be stated that the CMV was not a problem in this study. Later, the CFA was performed.

The four-factor structure established by Edwards et al., (2021) [4] was tested by using the confirmatory factor analysis (CFA) with maximum likelihood estimation [4]. Given that the Mardia coefficient showed a value of 98.16 and the critical ratio was 78.74, the sample can be considered non-normal multivariate. Hence, the authors applied bootstrapping with 5000 resamplings (95% CI) to solve non-normality. The result led to a first model: χ^2^ = 133.101; df = 48; χ^2^/df = 2.77; CFI = 0.96; IFI = 0.96; TLI = 0.96; RMSEA = 0.045 (90% Confidence Interval—CI90%: 0.036–0.054); SRMR = 0.037; AIC = 217.101; *p* < 0.001. Although the values of the fit indices were good, χ^2^/df had a high value. An investigation of the modification indices (MIs) led to a correlation errors between items 3 (My smile is an important part of my personal appearance) and 7 (I take pride in my teeth and gums). Thus, the new result was χ^2^ = 114,285; df = 47; χ^2^/df = 2.43; CFI = 0.96; IFI = 0.96; TLI = 0.95; RMSEA = 0.041 (90% Confidence Interval—CI90%: 0.031–0.050); SRMR = 0.034; AIC = 200.285; *p* < 0.001 (Table 1). As it is known, the χ^2^ is sensitive to a large sample size, and a statistically significant χ^2^ test is not uncommon [43]. The standardized factor loading was >0.40 in the case of all 12 items and, therefore, the latter can be retained according to the recommendations of Pituch and Stevens (2016) [44]. The lower factor loading was that of items 9 and 4 (0.44 and 0.47, respectively) and the highest was that of items 10 and 5 (0.80 and 0.88, respectively) (Appendix A).

### 3.3. Reliability and Descriptive Statistics

The analysis of descriptive statistics per factors showed that the highest average value was registered by the scales for Appearance (M = 13.97; SD = 1.71) and Retention (M = 13.47; SD = 1.81) (Table 2). As for reliability, two subscales, Appearance and Flossing, showed α and ω coefficients over 0.70. In addition, the entire scale registered good values, ω = 0.76 (95%CI—0.73–0.79) and α = 0.76 (95%CI—0.73–0.78) (Cronbach’s α if the item was deleted was between 0.72 and 0.76).

The high value of the consistency coefficients for the entire scale shows the fact that all 12 items of the scale measure the same construct. The Professional dental care scale showed a reasonably low–moderate value (α = 0.57; ω = 0.56), while the Retention subscale showed poor internal consistency (α = 0.46; ω = 0.46) (Table 2). The calculation of Cronbach coefficients in the case of subsamples of males and females showed that the latter are lower for the female subsample, except for the Flossing subscale: Professional dental care (α = 0.55—females; α = 0.56—males), Appearance (α = 0.66—females; α = 0.71—males), Flossing (α = 0.78—females; α = 0.74—males), Retention (α = 0.34—females; α = 0.54—males). As for the average scores of the items, it can be seen that the latter varied from 2.89 (item 2) to 4.84 (item 6) and 4.85 (item1), suggesting that the individuals in the Romanian sample valued a method such as flossing less, but they are still interested and they invest time and energy in how their teeth look (Table 3).

The values of skewness and kurtosis were >2 and >7, respectively [45], which means that the scores were not normally distributed; the highest values of skewness and kurtosis were the values of items 1 (It is important to me to keep my natural teeth) and 6 (I would rather get dentures than spend money to treat cavities or gum disease), which both entailed extreme affirmative reactions from the respondents.

### 3.4. Convergent Validity

Convergent validity was certified by the fact that the OHVS total score and the four subscales correlated with the instruments that assess constructs of oral health (Table 4). Thus, there were moderate negative correlations of the OHIP-14 with the OHVS total score (r = −0.16; *p* < 0.001) and the Professional (r = −0.14; *p* < 0.01), Flossing (r = −0.10; *p* < 0.01), and Retention (r = −0.15; *p* < 0.01) subscales. The exception was the Appearance subscale, which poorly correlated with the OHIP-14 score. Negative correlations were obtained between the OHVS (all the subscales) and the R-DBS (r range from −0.17 to −0.33; all at *p* < 0.01). Positive correlations were obtained between the OHVS (all the subscales) and the DNS (r between 0.11 and 0.37) and general health literacy (r between 0.19 and 0.34). The correlations between the OHVS subscales were moderate (r between 0.23 and 0.37), but their associations with the total score were high (r between 0.62 and 0.74). All the intercorrelations within the OHVS were <0.85 and higher than the correlations of the OHVS with the other tests, which shows the discriminant validity [38].

### 3.5. Discriminant Validity

At the same time, the Fornell–Larcker [40] criterion was used to test the discriminant validity of the scale. Thus, the Average Variance Extracted (AVE) was calculated based on λ and ε obtained in the CFA. The square root of the AVE in each construct was compared with its inter-construct correlation. If it was higher, then the discriminated validity was confirmed. Observing Table 5, it can be stated that the OHVS fulfills the criterion of discriminant validity.

### 3.6. Gender Differences

The construct validity of the scale was also verified by the gender effect. The results obtained through the Mann–Whitney U test confirmed the high scores obtained by the subsample of females in comparison with the male one for all four subscales: Professional dental care, Appearance, Flossing, and Retention, and for the OHVS total score (all at *p* < 0.001) (Table 6).

## 4. Discussion

The study focused on the translation of the OHVS and the assessment of its validity and reliability in the case of a Romanian adult population.

The OHVS authors concluded that the relevant values for oral health comprise four factors with implications on aspect and functionality: Professional dental care, Appearance, Flossing, and Retention of the natural teeth. The correlations between the OHVS and other measures of oral health constructs and oral health behaviors in the development and validation samples provided evidence on the convergent and competing validity. Under such conditions, the OHVS seems a useful and promising measure in epidemiologic and behavioral dental research [4].

Based on these considerations, for the cross-cultural validation of the OHVS, the authors resorted to a factorial structure analysis through CFA and selected the best-known tools in the oral health field, some already validated by the Romanian population, out of which some can be seen in the validation of the English version of the OHVS, as it has already been shown above: OHIP-14, R-DBS, DNS, GHL. After making sure that the social desirability did not affect the completion of the instruments, we proceeded to the CFA.

The confirmatory factorial analysis supported the structure of the four-factor scale and the integral presence of all 12 items. The CFA showed that the scale has good fit indices, and all the items have a loading factor over 0.40. Considering the replication of the four-factor structure of the scale and the good value of the goodness-of-fit indices, it was considered that the findings of the present study are in line with those obtained in the validation of the English version [4].

The comparison of the average scores of the subscales obtained in the current study with those obtained on the American sample [4] showed a hierarchy similar to the latter: Appearance and Retention of teeth had the highest values, followed by Professional dental care and, finally, Flossing, which suggests similar values regardless of the cultural model.

As for internal consistency, evidence was found for the general scale and for two subscales, Appearance and Flossing, which had good internal consistency, while the subscales Professional dental care and Retention of natural teeth had reduced consistency. This could be due to the fact that the subscales had a small number of items, three. On the other hand, as the literature explains, Cronbach´s α internal consistency coefficients are influenced by how many items a scale has [46]. When the number of items is below 10, the Cronbach’s α values are quite low [47]. Therefore, for a scale of three items, the value of coefficients is expected to be a moderate one.

As expected, the OHVS significantly correlates with all the instruments within the study that measure aspects related to oral health. The convergent validity proved that the OHVS and its subscales are negatively associated with the disbelief in dental services (R-DBS) and with negative oral health impacts (OHIP-14), although the subscale Appearance is poorly related, suggesting no convergence, and positively associated with oral self-care (DNS) and general health literacy expressed through the ability to understand and read medical materials (GHL). The findings are consistent with those of the OHVS development study [4]. Unlike the original study [4], in the present research, weak and moderate correlations were obtained between the OHVS and the other instruments used for convergent validity.

The correlations between the four factors of the OHVS showed that the latter are associated, but they measure different concepts. The highest correlation was between Appearance and Retention of teeth. A comparison with the original study in which the OHVS was developed and validated showed a similar result [4]. The discriminant validity was certified by the Fornell–Larcker criterion [40]. The values of the square root of the indicator AVE were higher than the inter-construct correlation coefficients for all factors.

The construct validity of the scale was also verified by the gender effect. The literature shows that women have more dental anxiety [48]; therefore, they frequent more dental care practices [49], and they have positive attitudes towards dental hygiene [50,51,52,53]. In addition to using dental floss more than men do, women trust the efficiency of the latter [54,55]. This pattern was noticeable in the present sample as well. As for gender differences, the results confirm prior data in the literature that show that women invest more in professional care and aspect, and they believe in the efficiency of flossing. The result is consistent with that of other pieces of research. For example, it is demonstrated that, while both men and women have the same level of knowledge about oral health, they do not have the same attitudes and behaviors. Women invest more in their aspect and beauty than men do; therefore, they tend to take better care of their teeth, to follow the doctors’ instructions, and to show up on time for appointments [54].

Research carried out on samples of Romanian subjects showed the fact that women take more measures with regard to their oral health precisely because they care more about oral health [8]. For women, their smile is a critical reason for which they resort to oral health services [56].

## 5. Limitations

Several limitations are worth mentioning. Although the data come from a relatively large sample, the latter is still a probabilistic one, which can make the results less representative. Secondly, although the age range greatly varies (the age bracket is 18 and 75), there is still a large number of young students (18–29 years old—58% and 30–35 years old—8%). The limitation provides the opportunity for further research, since subsequent studies could take into consideration the validation of the scale on specific populations, such as teenagers, emerging adults, elderly people, etc. In addition, there is a high number of women in the sample, which means that the results are relevant for this gender. The gender disproportion may be due to the receptivity of the female population in completing questionnaires that improve self-knowledge. Of course, this aspect limits the generalization of the results. Another possible limitation is related to the fact that the data regarding the frequency of visits to the dentist were not collected. Last, but not least, another aspect related to the sample comes from the level of education of the participants, who are mostly people with higher education. Clearly, this percentage of people with higher education does not reflect the actual situation in Romania.

Thus, there is the need for future research on samples that are equally distributed concerning gender and education level in order to have a more accurate validation of the Romanian version of the OHVS. Despite these limitations, the proof regarding the construct validity of the OHVS is a starting point for subsequent research regarding the examination of the OHVS properties. In this sense, in order to better understand the quality of the items and of the information provided by the OHVS, the authors consider that it is important to carry out subsequent research that should use the item response theory (IRT) analysis.

## 6. Conclusions

The study is the first to assess the psychometrical properties of the OHVS in a cultural model other than the one in which it was developed. The obtained findings provide certain evidence in favor of the cross-cultural validity of the scale. The OHVS is a psychometrically sound measure. The practical implications of the study are related to the need to use the scale in research on the values of oral health in epidemiological studies, and, on the other hand, its usage can contribute to the development of databases regarding oral health, which in Romania are underperformed. The study is likely to lead to knowledge of oral health values in order to prevent illness and to promote the patient’s well-being, but also to help change the perception of the importance of oral health education.

## Figures and Tables

**Table 1:** Goodness-of-fit of confirmatory factor analysis model.

Models	χ^2^	df	χ^2^/df	CFI	IFI	TLI	RMSEA (CI_90%_)	SRMR	AIC
M1 four factors without correlated errors	133.101	48	2.77	0.96	0.96	0.96	0.045 (0.036–0.054)	0.037	217.101
M1 four factors with correlated errors	114.285	47	2.43	0.97	0.97	0.95	0.041 (0.031–0.050)	0.034	200.285

Note: CFI: comparative fit index; IFI: incre-mental fit index; TLI: Tucker–Lewis index; RMSEA: root mean square error of approximation; SRMR: standardized root mean square residual; AIC: Akaike’s Information Criterion.

**Table 2:** Means, standard deviations, and reliabilities (*n*= 869).

Factors	M	S.D.	Min–Max	α	ω
Professional dental care	11.72	2.70	1–5	0.57	0.56
Appearance	13.97	1.71	1–5	0.72	0.72
Flossing	8.93	3.30	1–5	0.78	0.79
Retention teeth	13.47	1.81	1–5	0.46	0.46
OHVS total score	48.12	6.75	19–60	0.76	0.76

Note: M = mean; S.D. = standard deviation; OHVS: Oral Health Values Scale.

**Table 3:** The descriptive statistics per items.

Items	M	S.D.	Skewness	Kurtosis	α if Item Deleted
1	4.85	0.45	−3.70	15.89	0.75
2	2.89	1.31	0.11	−1.00	0.74
3	4.70	0.67	−2.79	8.59	0.75
4	4.07	1.14	−1,12	0.35	0.75
5	2.99	1.28	0.02	−1.00	0.72
6	4.84	0.63	−4.60	21.71	0.75
7	4.60	0.78	−2.15	4.62	0.74
8	4.07	1.15	−1.07	0.09	0.73
9	3.79	1.28	0.72	−0.63	0.75
10	3.05	1.35	−0.01	−1.13	0.73
11	3.57	1.39	−0.56	−0.98	0.75
12	4.68	0.64	−2.26	5.57	0.74

Note: M = mean; S.D. = standard deviation.

**Table 4:** Intercorrelations between the OHVS and other measures.

Variables	1	2	3	4	5	6	7	8
1. OHVS total	-							
2. Professional dental care	0.74 ***	-						
3. Appearance	0.62 ***	0.31 ***	-					
4. Flossing	0.76 ***	0.34 ***	0.30 ***	-				
5.Retention	0.62 ***	0.37 ***	0.36 ***	0.23 ***	-			
6. OHIP-14	−0.16 ***	−0.14 ***	−0.07 *	−0.10 **	−0.15 ***	-		
7. DNS	0.37 ***	0.21 ***	0.32 **	0.36 ***	0.11 ***	−0.11 **	-	
8. R-DBS	−0.33 ***	−0.36 ***	−0.19 ***	−0.17 ***	−0.21 ***	0.20 **	−0.13 ***	-
9. Health literacy	0.34 ***	0.27 ***	0.23 ***	0.24 ***	0.19 **	−0.14 **	0.19 **	−0.31 ***

Note: *** *p* < 0.001; ** *p* < 0.01; * *p* < 0.05.

**Table 5:** Discriminant validity: Fornell–Larcker criterion.

Factors	1	2	3	4
Professional dental care	0.55 *			
Appearance	0.31	0.64 *		
Flossing	0.34	0.30	0.75 *	
Retention	0.37	0.36	0.23	0.51 *

Note: * Square root of AVE value for each factor.

**Table 6:** The effect of gender differences.

Factors	Mean Rank		Mann–Whitney U	Z
	Males	Females		
Professional dental care	375.02	460.02	62,505.00	4.56
Appearance	328.77	478.78	50,894.50	9.13
Flossing	355.22	468.05	57,334.50	6.02
Retention	380.34	457.87	63,840.50	4.29
OHVS total score	329.38	478.53	51,049.00	7.94

Note: All the results are at *p* < 0.001.

**Figure A1:**
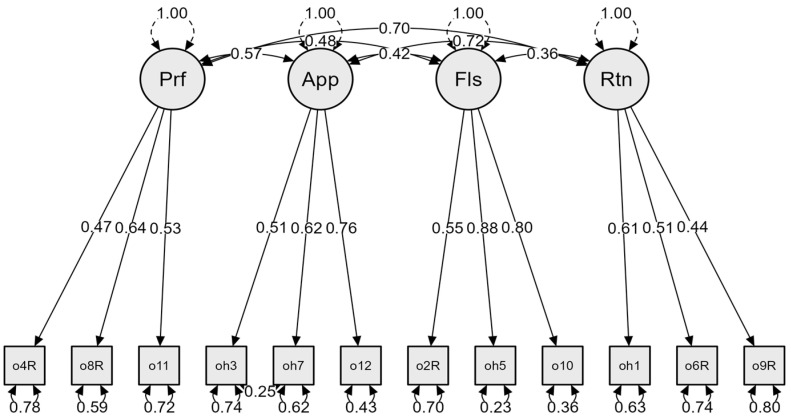
Confirmatory factor analysis of the OHVS with the standardized factor loading. Reversed items are denoted by R. Prf: professional, App: appearance, Fls: flossing, RTN: retaining.

## Data Availability

The data presented in this study are available from the corresponding authors upon reasonable request.
